# Effect of Different Cooking Methods on Proton Dynamics and Physicochemical Attributes in Spanish Mackerel Assessed by Low-Field NMR

**DOI:** 10.3390/foods9030364

**Published:** 2020-03-21

**Authors:** Shan Sun, Siqi Wang, Rong Lin, Shasha Cheng, Biao Yuan, Zhixiang Wang, Mingqian Tan

**Affiliations:** 1 School of Food Science and Technology, Dalian Polytechnic University, Dalian 116034, Liaoning, China; wiseshansun@163.com (S.S.); wangsiqi93dpu@163.com (S.W.); lr131789@163.com (R.L.); chengshasha880321@126.com (S.C.); 2 National Engineering Research Center of Seafood, Dalian 116034, Liaoning, China; 3 Collaborative Innovation Center of Seafood Deep Processing, Dalian 116034, Liaoning, China; 4 Engineering Research Center of Seafood of Ministry of Education of China, Dalian 116034, Liaoning, China; 5 College of Engineering/National R&D Center for Chinese Herbal Medicine Processing, China Pharmaceutical University, Nanjing 211198, Jiangsu, China; yuanbiao@cpu.edu.cn (B.Y.); chinawzx@sohu.com (Z.W.)

**Keywords:** spanish mackerel, protons migration, protein oxidation, lipid oxidation, cooking methods, texture

## Abstract

The states of protons within food items are highly related to their physical attributes. In this study, the effect of cooking methods including boiling, steaming, roasting and frying on proton dynamics, physicochemical parameters and microstructure of Spanish mackerel was assessed by low-field nuclear magnetic resonance (LF-NMR) and magnetic resonance imaging (MRI) techniques. The treatment of cooking resulted in a significant reduction of proton mobility and declined freedom of protons. The state changes of protons can be monitored easily in an intuitive and non-destructive manner during various cooking process. The treatments of boiling, steaming, roasting and frying resulted in different cooking loss and similar water-holding capability. A significant increase of total carbonyl content and thiobarbituric acid reactive substances was found, while a decrease of the values for free thiols and surface hydrophobicity was observed. The analysis of circular dichroism spectroscopy and cryo-scanning electron microscopy showed significant structural change. The correlation coefficients of R_cal_^2^ and R_cv_^2^ from partial least squares (PLS) regression models were more than 0.980, suggesting good correlation between LF-NMR data and hardness, resilience, springiness, chewiness, gumminess, and adhesiveness. Good recoveries and a relatively small coefficient of variation (CV) were obtained from the PLS regression models, indicating good reliability and accuracy in predicting texture parameters for mackerel samples.

## 1. Introduction

Spanish mackerel, an important fish, contains high-quality protein and polyunsaturated fatty acids, which can reduce the risk of heart disease and maintain human health [1]. In general, the Spanish mackerel is cooked before eating to prevent infection from pathogenic bacteria, and gains enhanced flavor and taste. Traditional cooking methods include boiling, steaming, roasting and frying. During cooking, a series of physicochemical reactions occur within the flesh. This can cause significant water loss, protein denaturation and lipid oxidation [2]. It is well known that, water, an important ingredient in fish, accounting for about 80% of the total mass, plays a critical role in cooking quality and texture characteristics. However, it is a challenge to characterize the water dynamics, protein denaturation and lipid oxidation during various thermal processes of Spanish mackerel. Therefore, it is vital to determine the effect of different cooking methods on protein and lipid state changes in Spanish mackerel.

The states of protons from water, proteins, lipid and polysaccharide are highly related to fish muscle destruction and protein denaturation during thermal processes. Most of the protons coming from the water within the fish meat can be reflected through monitoring relaxation parameters by radio frequency with the low-field nuclear magnetic resonance (LF-NMR) and magnetic resonance imaging (MRI) techniques [3]. The protons are chemically exchanged back and forth from water to macromolecules in fish meat, so that their transverse component of the magnetization vector can be measured by the spin-to-spin relaxation time (*T*_2_). Different food samples have different relaxation time *T_2_*, and therefore the parameters of *T*_2_ can reflect the change of physical attributes [4]. In addition, the MRI can provide visualized internal information of the food items during processing and storage [5]. At present, the non-destructive LF-NMR and MRI technology has exhibited prominent superiority in assessing food proton change during various food-processing methods, such as rehydration of sea cucumbers [6], drying of surf calm and chicken [7], cooking change of striped snakehead fish, and evaluating frozen pork [8,9]. However, to the best of our knowledge, there is not sufficient knowledge about the effects of cooking methods on proton changes in fish meat during thermal food processing. Therefore, it is necessary to develop a fast and efficient approach to analyze the correlation among protons, structural and textural changes, thereby providing a possibility for the substitution of instrumental methods in determining texture properties in a non-destructive manner.

The purpose of this study was to investigate the effect of four kinds of thermal cooking methods (boiling, steaming, roasting, frying) on the proton states from water, protein and lipid oxidation of Spanish mackerel by the LF-NMR and MRI technique. The dynamic proton migration of Spanish mackerel was recorded to clarify the mechanism of proton change during the different cooking processes. The ^1^H LF-NMR *T*_2_ relaxation time combined with MRI images were used to assess the water state and migration. Meanwhile, physicochemical changes such as texture, color parameters, cooking loss and water-holding capacity (WHC) were determined in different thermal processes. The microstructure of the Spanish mackerel was also analyzed by cryo-scanning electron microscopy. In addition, the correlation between LF-NMR data and texture parameters was measured for Spanish mackerel in various processing methods.

## 2. Materials and Methods 

### 2.1. Cooking of Fish Samples 

Spanish mackerels were bought from a food market (Paoaizi, Dalian China), which were kept in a cooling container and transported to the laboratory. After thawing at room temperature, the mackerel samples were sliced into 2.0 × 2.0 × 1.5 cm pieces, and randomly divided into five groups: boiling, steaming, roasting, frying and control group, respectively. For the boiling group, the fish samples were directly dropped in boiling tap water and cooked for 5 min. For the steaming group, the fish samples were put in a steamer above boiling water and steamed for 5 min. For the roasting group, the fish samples were put in an electronic oven at 200 °C for 20 min. For the frying group, the fish samples were put in frying palm oil at 180 °C for 5 min. 

### 2.2. H Low-Field Nuclear Magnetic Resonance (LF-NMR) and Magnetic Resonance Imaging (MRI) Analysis 

The transverse relaxation time (*T*_2_) was measured by an MesoMR23-060V-I NMR analyzer (Suzhou Niumag Analytical Instrument Corporation, Suzhou, China) with a permanent magnet having resonance frequency 21 MHZ at 32 °C. The fish meat sample was placed on a 60 mm diameter sample chamber for LF-NMR measurement. The Carr–Purcell–Meiboom–Gill (CPMG) decay signal was collected with pulses of 90° and 180° at 26 and 52 μs. The τ-value was set 200 μs and the number of echoes (NECH) was 8000 which were recorded in a sixteen repetition with repetition time 4500 ms. The relaxation curves M(t) were fitted by the following equation:

(1) $$M(t)=\sum_{n=1}^{N}M_{0,n}\exp\left(\frac{-t}{T_{2,n}}\right)+e\left(t\right)$$

where M(t) represents the residual magnetization at decay time t, M_0,n_ is the magnitude parameter of the n^th^ exponential, *T*_2,n_ is the corresponding transverse relaxation time constant, and e(t) is the residual error [10].

The MRI images of samples were acquired on a MesoMR23-060V-I NMR analyzer too. *T*_1_ weighted images was recorded using spin-echo imaging sequence with field of view (FOV) of 100 mm × 100 mm, slice gap: 2.0 mm, slice width: 2.6 mm, offset slice: 26.4 mm, average: 2, phase size: 192 and read size: 256. The echo time (TE) was 20 ms and repetition time (TR) was 500 ms. OsiriXLite software (version 7.0.4, Pixmeo, Geneva, Switzerland) was used for analysis of the MRI images.

### 2.3. Cooking Loss and WHC Measurements

The cooking loss of fish pieces was calculated by the weight before and after they were boiled, steamed, roasted and fried. Cooking loss = (W_initial_ − W_final_)/W_initial_ × 100%. The WHC was measured by placing the fish meat samples in a centrifugation tube. The fish samples were weighted before and after centrifugation at 5000 g for 5 min. The WHC was calculated by the equation [11]:

WHC = (W_b_ − W_a_)/W_b_ × 100% (2)

where W_b_ is the weight of fish sample before centrifugation, W_a_ is the weight of fish sample after centrifugation.

### 2.4. Color Analysis

A UltraScan PRO color analyzer (Hunter Associates Laboratory, Inc. Reston, VA, USA) was used for the analysis of fish color using a D65 light as the irradiation source. The parameters of a* (redness), L* (lightness), b* (yellowness) and whiteness were used to evaluate the difference in color change. Five groups were tested for each processing methods. Whiteness was calculated using Equation [12]:

W (whiteness) = 100 − [(100 − L)^2^ + a^2^ + b^2^]^1/2^ (3)

### 2.5. Textural Profile Analysis (TPA)

Textural profile analysis (TPA) parameters were measured before and after the treatment of boiling, steaming, roasting and frying with a TA. XT Plus Texture Analyzer (Stable MicroSystems, London, UK) by cutting fish samples into 1.5 × 1.5 × 2 cm slices. The pre-speed was 2.0 mm/s, test speed and post-speed was 1.0 mm/s, respectively. Each sample was measured at a compression level of 30% with a relaxation time between the two compressions 5 s.

### 2.6. Extraction of Myofibrillar Protein (MP) from Spanish Mackerel Meat

A total of 6 g cooked mackerel meat was homogenized at 9000 rpm for 1 min with five-fold volume of 0.05 M NaCl-tris buffer, and centrifugated at 10,000× *g* for 10 min at 4 °C. After removing the supernatant, the precipitate was homogenized and centrifugated with 0.6 M NaCl-tris buffer to obtain supernatant. The MP concentration in supernatant was measured by the Biuret method with serum albumin [13].

### 2.7. Surface Hydrophobicity of the MP

When the MP molecule was denatured, the internal hydrophobic amino acid was exposed on the surface and had the ability to bind to 8-anilino-1-naphthalenesulfonic acid (ANS), which can exhibit fluorescence [14]. The relative fluorescence intensity was positively correlated with the surface hydrophobicity [15]. The surface hydrophobicity (S0) of MP was measured by the method according to the previous report. All measurements were determined in triplicate.

### 2.8. Total Carbonyl Content

A total 2 g of fish meat was homogenized with 10 mL phosphate buffer (0.02 M, pH 6.5) and centrifuged at 13,000 r/min for 10 min. The supernatant with a volume of 0.4 mL was collected and added to 0.4 mL of 2,4-dinitrophenylhydrazine (DNPH, 10 mM) in 2 M HCl. The same volume of 2 M HCl buffer without DNPH was used as a control. The mixture was placed in dark condition for 1 h, vortexed every 10 min. and then precipitated with 0.5 mL of 20% (*w*/*v*) trichloroacetic acid. The precipitate was washed with ethanol-ethyl acetate (1:1, *v*/*v*) for three times, and dissolved in 6 M guanidine hydrochloride (GH) at 30 °C for 15 min. The total carbonyl content of the fish sample was measured by recording the absorbance of GH supernatant 370 nm [16].

### 2.9. Free Thiols Measurement

The measurement of free thiols oxidation was modification according to the method of Ellman [17]. The extracted MP concentration was diluted to 2 mg/mL, 2 mL diluted MP was added in phosphate buffer solution (pH 7.4, 8 mol urea, 0.1 mol sodium dodecyl sulfate) and 0.5 mL of 10 mmol 5,5′-dithiobis-(2-nitrobenzoic acid) (DTNB) for 15 min at room temperature (about 25 °C). After reaction, the absorbance at 412 nm was recorded against a blank solution of the same concentration MP without DTNB. The results were expressed as nmol of free thiols per milligram of MP.

### 2.10. Lipid Oxidation

The oxidative degradation of lipids in fish meat was evaluated by the thiobarbituric acid reactive substances (TBARS) assay [18]. A total of 1 g cooked fish sample was added in 5 mL of stock solution containing 0.375% thiobarbituric acid, 0.25 M HCl and 15% trichloroacetic acid. The fish samples were heated at 100 °C for 20 min followed by a centrifugation at 8000 rpm for 15 min at 4 °C. The absorbance at 532 nm was measured for the supernatant using an ultraviolet–visible (UV–vis) spectrophotometer. The malondialdehyde (MDA) concentration was as described in the previous work [18].

### 2.11. Cryo-Scanning Electron Microscopy (Cryo-SEM)

The cooked fish sample was cut into slices with a size of 0.5 × 0.5 × 0.5 cm for cryo-scanning electron microscopy (Cryo-SEM) analysis. The fish slices were fixed on the supporter, added in nitrogen (−210 °C) and then transferred to a vacuum chamber (PP3010T, Oxford, UK) at −140 °C. After being sublimated at −90 °C for 10 min, the fish sample was analyzed by a scanning electron microscope (SU8010, Hitachi Co, Tokyo, Japan) with an accelerating voltage of 1.0 kV [19].

### 2.12. Circular Dichroism (CD)

The MP supernatant was further purified using a dialysis bag with a molecular weight cut-off 3.5 kilodalton (KD) against deionized water to remove the impurities for 24 h at 4 °C. Then the supernatant was diluted to 0.2 mg/mL for circular dichroism (CD) analysis. A J-1500 CD spectropolarimeter (JASCO. Tokyo. Japan) was used to measure the CD spectra at the wavelength range of 250–190 nm with a scan speed of 50 nm/min. A cuvette with path length of 1 mm was used for the measurements. The secondary structural contents of MP were calculated according to the previous method [20]. 

### 2.13. Statistical Analysis 

One-factor analysis of variance (ANOVA) was applied for each parameter by the commercial SPSS 20.0 software with a statistical significance at *p* <0.05 (SPSS Inc., Chicago, IL, USA). Partial least squares (PLS) regression models were performed using the software Unscrambler 9.7 (CAMO Software Inc., Montclair, NJ, USA). The CPMG decay signals of fish samples were correlated by the PLS regression model to develop a multivariate linear regression model.

## 3. Results

### 3.1. Proton Dynamics during Boiling, Steaming, Roasting, Frying Assessed by LF-NMR

**Figure 1:**
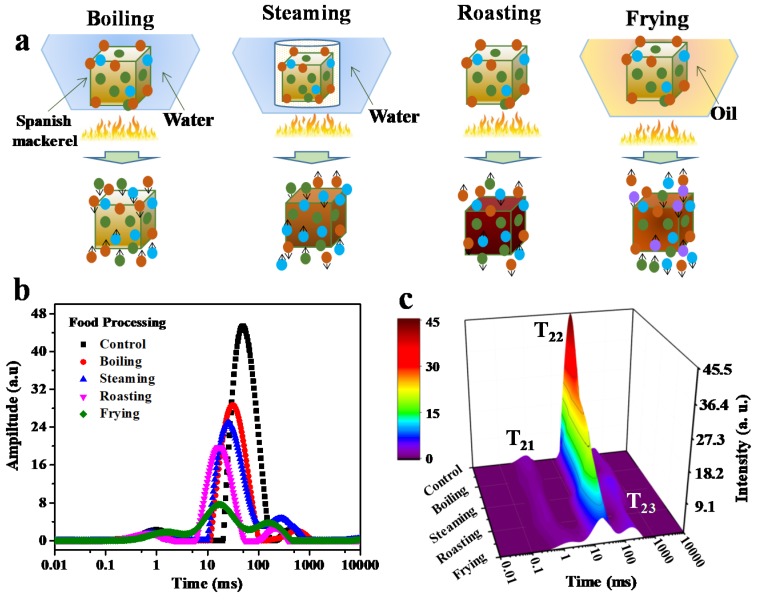
Schematic illustration of proton changes (**a**), *T*_2_ relaxation time distribution curves (**b**) and *T*_2_ relaxation spectra in 3D color map surface image (**c**) of Spanish mackerel before and after the treatment of boiling, steaming, roasting, frying (*T*_21_, bound water, *T*_22_, immobilized water, *T*_23_, free water). Control sample is the fish meat before heating treatment.

Different thermal food processing can result in different proton changes, thus affecting their physicochemical properties. As shown in Figure 1a, four types of thermal processes, such as boiling, steaming, roasting and frying, were applied to the mackerel meat to investigate the food processing on proton change. There are mainly three peaks in the *T*_2_ relaxation curves. The shortest relaxation time *T*_21_ from 0.1 to 10 ms is assigned to protons attached to the biomacromolecules, the prominent component *T*_22_ between 10 and 200 ms is due to immobilized water trapped in the myofbrillar network, and the third population, *T*_23_, with a relaxation time of 200–1000 ms corresponds to extra-myofbrillar free water [21]. In contrast to the relaxation profile of sample before cooking, the values of *T*_21_ fluctuated in the range of 0.66–1.44 ms, while the *T*_22_ and *T*_23_ relaxation time showed an obvious blue shifting, from 51.04 to 17.43 ms, and from 429.96 to 142.94 ms, respectively, indicating a reduced proton mobility and declined freedom of the protons (Figure 1b, Table 1). Plenty of water was lost in the fish meat during thermal processing, thereby the remaining protons were restrained in the cooked meat resulting in a reduced mobility. Among the four types of food-processing method, frying caused the most significant change to the fish samples, and the *T*_22_ peak area (*A*_22_) significantly reduced from 9577.17 to 1707.53 per gram (Table 1). The cooking methods caused *T*_22_ water release in a descending order: boiling, steaming, roasting and frying, and frying led to the most water loss among the four types food processing. Instead of the reduction of peak area, however, the *T*_23_ peak area (*A*_23_) showed an increased trend for all the cooking methods. This demonstrated that the cooking media like water and oil possibly permeated the fish meat during boiling, steaming and frying, and the roasting method led to the slightest increase for *A*_23_ due to the protons expelled from *A*_22._ No protons from the cooking media were involved in the roasting process (Table 1), therefore, the protons within the fish meat were significantly driven out from the protein network. The *T*_2_ relaxation spectra in 3D color map surface image further shows the proton change in the mackerel meat during different food thermal processing (Figure 1c). The relaxation peak position and area showed a successive variation and the result was more intuitive for comparison.

**Figure 2:**
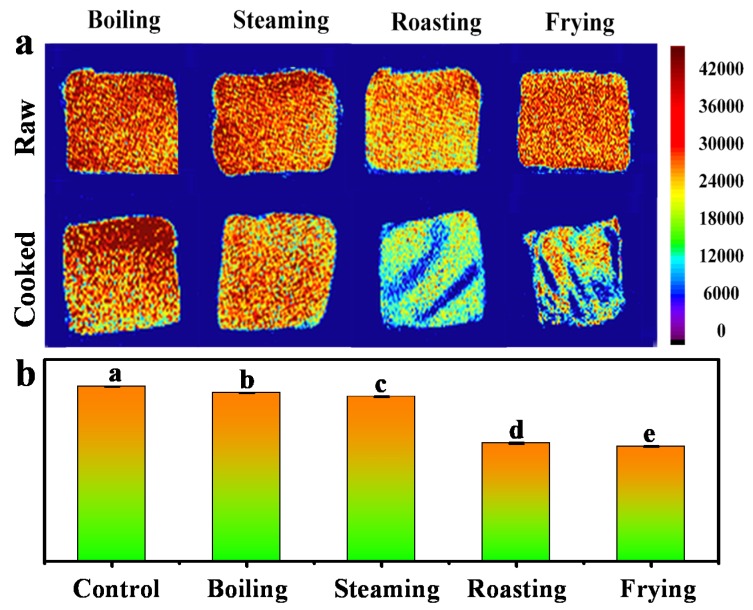
(**a**) *T*_1_ weighted magnetic resonance images of fish samples subjected to different cooking methods, (**b**) the corresponding histogram of quantitative signal intensity. Control is sample without heating treatment. Different letters indicate significant differences between groups (*p* < 0.05).

MRI, a fast, direct and non-invasive technique, has been used for food item analysis during food progressing [22]. The MRI images in Figure 2a display the tomography of the mackerel samples before and after being cooked by boiling, steaming, roasting and frying methods. The red color represents higher density while blue color refers to lower density. In contrast to the raw fish meat, the enhancement of the cooked mackerel samples exhibited a lower intensity, especially for roasting and frying groups, which displayed an obvious stripe pattern, suggesting significant change of the proton states during roasting and frying. Similar result was also found in the proton change of MRI with turbot flesh during boiling, stewing and frying processes [23]. Figure 2b clearly shows the relative intensity of *T*_1_ weighted images for mackerel samples, and a significant decrease was observed among the groups of boiling, steaming, roasting and frying. The decrease of MRI intensity for mackerel samples after roasting and frying demonstrated that they were more drastic cooking methods than boiling and steaming in changing the proton states. With the use of the MRI technique, the inner structure change of mackerel meat can be easily visualized and the detailed information of the proton distribution can be obtained in evaluating the change of mackerel meat in a non-destructive manner. 

### 3.2. Cooking Loss, Water-Holding Capacity Analysis 

**Figure 3:**
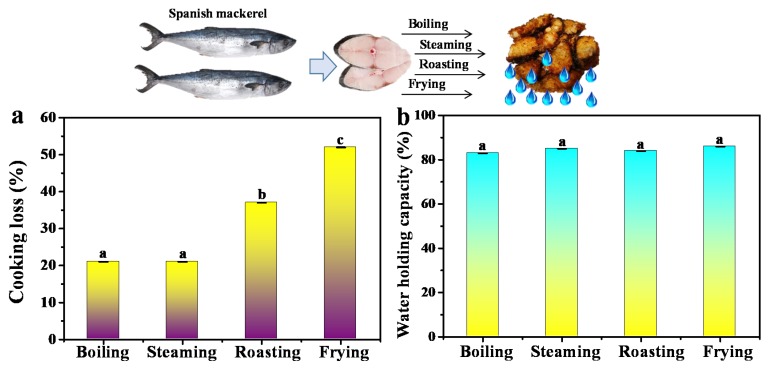
(**a**) Cooking loss of Spanish mackerel after the treatment of boiling, steaming, roasting and frying, (**b**) water holding capacity of Spanish mackerel after the treatment of boiling, steaming, roasting and frying. Different letters indicate significant differences between groups (*p* < 0.05).

Thermal food processing can result in cooking loss of the fish meat, in which there is plenty of loss of components including water, lipid, peptide and other flowable small molecules. As shown in Figure 3a, the cooking loss of mackerel samples after boiling and steaming was about 21%, while the cooking loss of roasting and frying was 37% and 52%, respectively. The cooking loss in the roasting and frying groups declined more significantly than that of boiling and steaming. The protons in mackerel samples were largely dispelled from the bio-macromolecules and released to the surrounding environment. As for frying, cooking oil can reach much higher temperatures than boiling water at normal atmospheric pressure, and the weight loss of fried mackerel samples was much larger than that of the roasting group. This was consistent with the results of LF-NMR analysis, demonstrating that roasting and frying might result in serious damage of the fish meat structure and induce significant cooking loss. Moreover, WHC were 83%, 85%, 84%, 86% (Figure 3b), respectively, for the samples after boiling, steaming, roasting and frying. The WHC did not change much, indicating that cooked fish samples maintained similar binding force for water. 

### 3.3. Color Analysis

**Figure 4:**
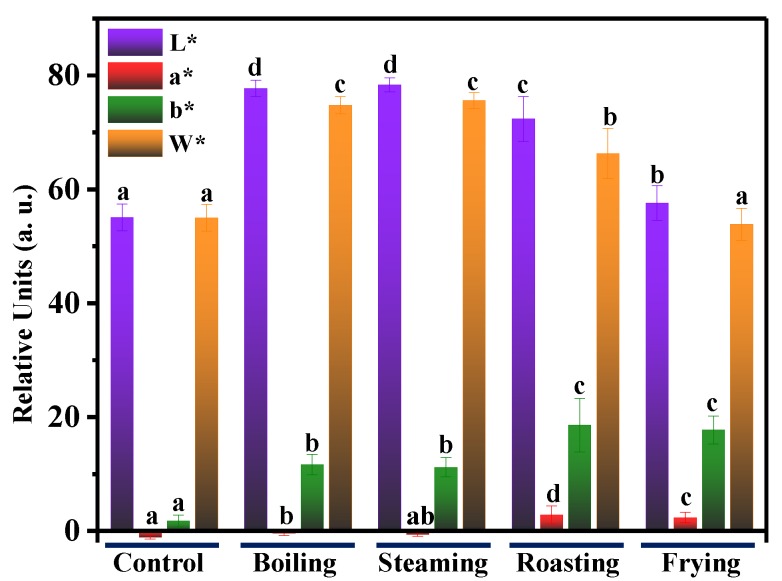
Lightness (L*), redness (a*), yellowness (b*) and whiteness (W*) of fish samples before (control) and after treatment of boiling, steaming, roasting and frying. Different letters indicate significant differences between groups (*p* < 0.05).

Color can reflect the appearance of food samples and is highly dependent on food processing [24]. Figure 4 shows the color parameters of mackerel samples after the treatment of boiling, steaming, roasting and frying. The L* values show a significant increase from 55.06 to 77.73, 78.36, 72.36 and 57.57, for boiling, steaming, roasting and frying, respectively. The increase suggested that the food processing turned the fish meat sample brighter than the control. This was possibly due to the water loss, protein denaturation and rearrangement of the protein after food processing, and thus increased the proportion of light reflection from the mackerel surface [19]. The a* value increased significantly in roasting and frying groups, but not in boiling and steaming groups, which was attributed to the protein degeneration and oxidation by Maillard reaction occurred in roasting and frying. The yellowness b* representing the degree of lipid oxidation also displayed an increasing trend, indicating that the high heating temperature of roasting and frying resulted in a larger b* value than boiling and steaming. By contrast, the whiteness values W* of roasting and frying were significantly lower than those of boiling and steaming.

### 3.4. Protein and Lipid Oxidation Characterization

Table 2 shows the physical attributes such as total carbonyl, free thiols, surface hydrophobicity and TBARS of the mackerel samples before and after treatment of boiling, steaming, roasting and frying. The total carbonyl of frying increased largely than those of boiling, steaming, roasting and control. The free thiols decreased significantly from 4.62 to 1.18, 1.02, 0.87, and 0.57, respectively, for boiling, steaming, roasting, and frying. Higher carbonyl content and lower free thiols value mean a higher degree of protein oxidation [25]. The main reason for the change was that high temperature caused protein oxidation and complexes of malondialdehyde were formed [26], which agreed with the color results. Additionally, the surface hydrophobicity, which represented the relative content of hydrophobic residues on the surface of protein molecules, decreased dramatically from 54.89 to 15.76 and 14.88, for roasting and frying. These values were lower than 19.34 and 20.67 for boiling and steaming, suggesting that the folded protein molecules began to degrade and some hydrophilic groups were exposed after heating, resulting in the decrease of surface hydrophobicity. Li et al. [27] reported that the surface hydrophobicity of protein isolate decreased at pH 9.0 after citric acid crosslinking due to the shielding effect of a large negative charge on proteins. In addition, protein denaturation often accompanies lipid oxidation [28], and the main indicator of lipid oxidation, TBARS, directly reflects the content of secondary oxidation product generated by lipid oxidation [29]. The TBARS values of boiling, steaming, roasting, frying groups increased from 0.17 (control) to 0.26, 0.36, 0.64, and 0.34 mg/kg, respectively. The TBARS value of roasted samples was significantly higher than that of the control group, reflecting that the oxidation of mackerel muscle was promoted at high temperature. However, the TBARS value of the fried sample was much lower than that of roasting because the higher temperature of frying oil could boost the TBARS reaction which decreased the content of TBARS. A similar result was also observed from lipid oxidation of *Acipenser gueldenstaedtii* [30].

### 3.5. CD Analysis

**Figure 5:**
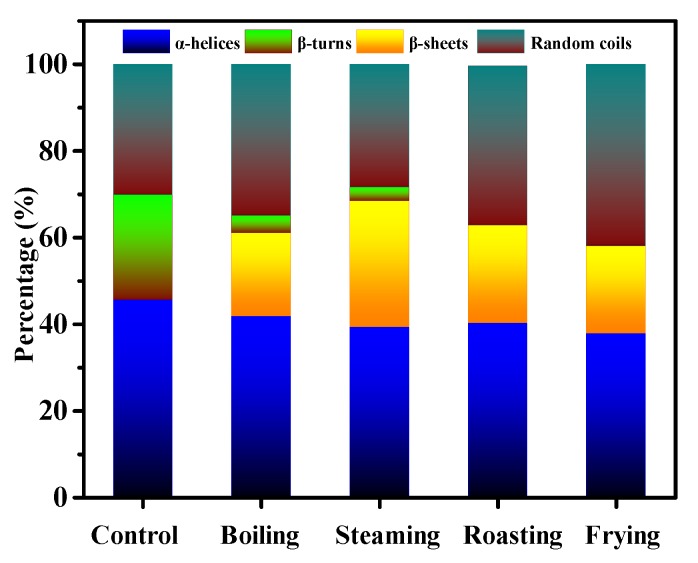
Effect of different cooking methods on the relative contents of α-helices, β-turns, β-sheets and random coils in MP of Spanish mackerel.

The CD spectra can reflect the change of secondary structure of fish protein after various cooking treatments [31]. Figure 5 shows the percentage of α-helices, β-turns, β-sheets and random coils of the fish meat samples before and after boiling, steaming, roasting and frying. The relative content of α-helices decreased after cooking of the fish MP. The proportion of β-turns significantly decreased, accompanied by the emergence of a large percentage of β-sheets. More specifically, β-turns disappeared completely in the MP after roasting and frying, while the β-turns in the MP was about 3.7% after boiling and steaming, indicating that high-temperature cooking resulted in drastic transition of β-turns. In addition, an increased proportion of random coils was observed for the boiling, roasting and frying groups. Bier et al. [32] proved that the protein of blood meat underwent structural rearrangement during heating and led to a lower α-helices and higher β-sheets content. This was consistent with the result of Spanish mackerel detected by the CD method. 

### 3.6. Cryo-SEM Spectra Analysis

**Figure 6:**
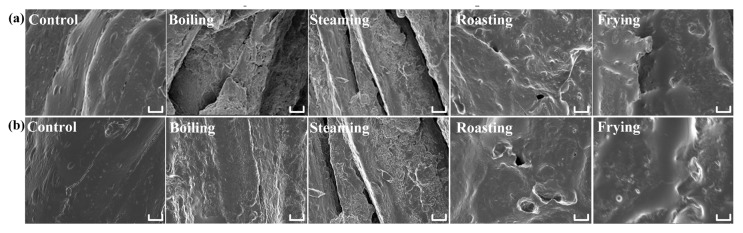
Cryo-scanning electron microscopy (cryo-SEM) images of microstructures of fish samples after boiling, steaming, roasting and Figure 600. b, × 1000). Control is the fish sample without heating treatment. Scale bar = 20 μm.

Cryo-SEM can provide more information about microstructure change for MP in the cooked mackerel meat. The Cryo-SEM images (Figure 6) of mackerel meat before (control) and after boiling, steaming, roasting and frying treatments show that heating process significantly changed the microstructure of the fish meat. The surface of the raw sample was smooth and no folded or cracked structures were observed (control). In contrast, obvious breakage was found in the fish sample after boiling and steaming treatment. The protein fibers broke into pieces and formed deep trenches, revealing the occurrence of protein denaturation. As for the roasted fish sample, spherical holes were found and the proteins of the fish sample stuck together without any fault zone. Frying led to shrinkage of the protein and infusion of frying oil, which was possibly caused by water loss and protein denaturation after frying [33]. The results revealed that different cooking methods resulted in different microstructural changes in the cooked fish meat samples.

### 3.7. Principal Component Analysis (PCA) of NMR Parameters 

**Figure 7:**
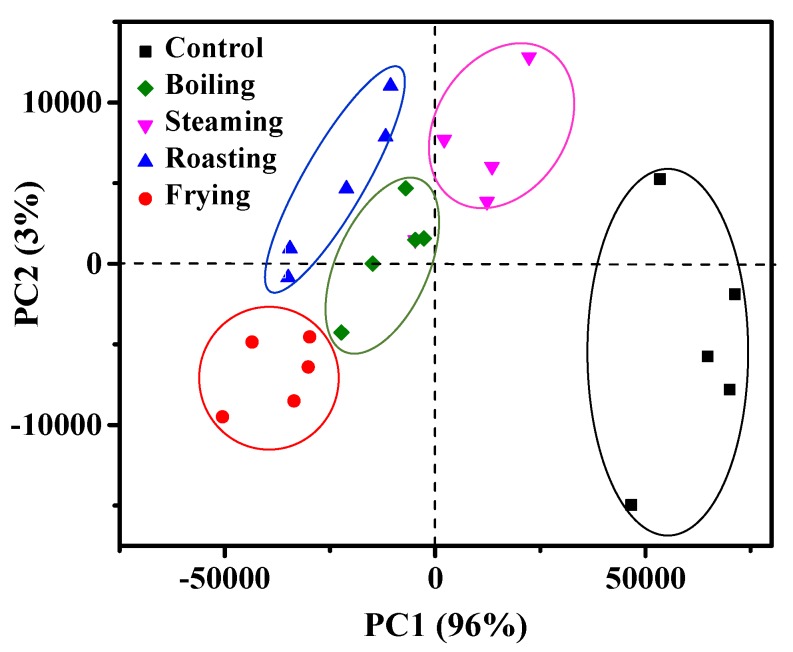
Principal component PC1 and PC2 scores plot from principal component analysis of fish samples with boiling, steaming, roasting, frying. Control is sample without heating treatment.

From the above analysis, we noted that different cooking methods led to significant changes of the fish samples. As a powerful statistical technique, principal component analysis (PCA) is an extensively used method in the field of vector algebra chemometrics for multivariate data sets analysis [34]. The mackerel samples after the treatments of boiling, steaming, roasting and frying were clearly discriminated using the PCA combined with LF-NMR spectra data (Figure 7). The mackerel samples before and after treatment of boiling, steaming, roasting and frying are well separated in Figure 7. The PC1 explained 96% of the total change of proton relaxation for *T*_2_, while the PC2 explained 3% of the total change. The first two main components (PCs) of the samples after boiling, steaming, roasting and frying treatment explained 99% of the total variation. These results showed that the mackerel samples with different cooking methods could be identified with the combination of LF-NMR and PCA. 

### 3.8. Texture Profile Analysis

The texture parameters of hardness, resilience, springiness, chewiness, gumminess and adhesiveness of mackerel meat before and after boiling, steaming, roasting, and frying treatment are shown in Table 3. Hardness refers to the magnitude of external force when the food sample reaches a certain deformation [35]. It can be seen that the hardness showed a significant increase after cooking, especially for the samples after roasting and frying. This was probably due to the dramatic cooking loss and protein denaturation as mentioned above. The change of resilience was not obvious compared with other parameters, and a significant increase was only observed for the samples after roasting. Moreover, springiness refers to the ratio of the height of the second compressed sample to the first one after the sample was deformed [36]. Meanwhile, the springiness of mackerel samples had an obvious increasing trend upon roasting and frying, which might be due to the increase of substances produced by lipid oxidation and polymerization during roasting and frying. Chewiness is the energy required to chew a sample in a stable state [37], and the change of chewiness is positively correlated with hardness. In the frying group, the chewiness was significantly higher than that of other cooking methods. Gumminess represents the property of being cohesive and sticky, which showed a significant increase after the treatment of boiling, steaming, roasting, and frying. Similar to resilience, the change of adhesiveness was not obvious compared with other parameters. All the results revealed that different cooking methods resulted in different changes in the textural properties for the mackerel meat.

### 3.9. Partial Least Squares (PLS) Regression Models

**Figure 8:**
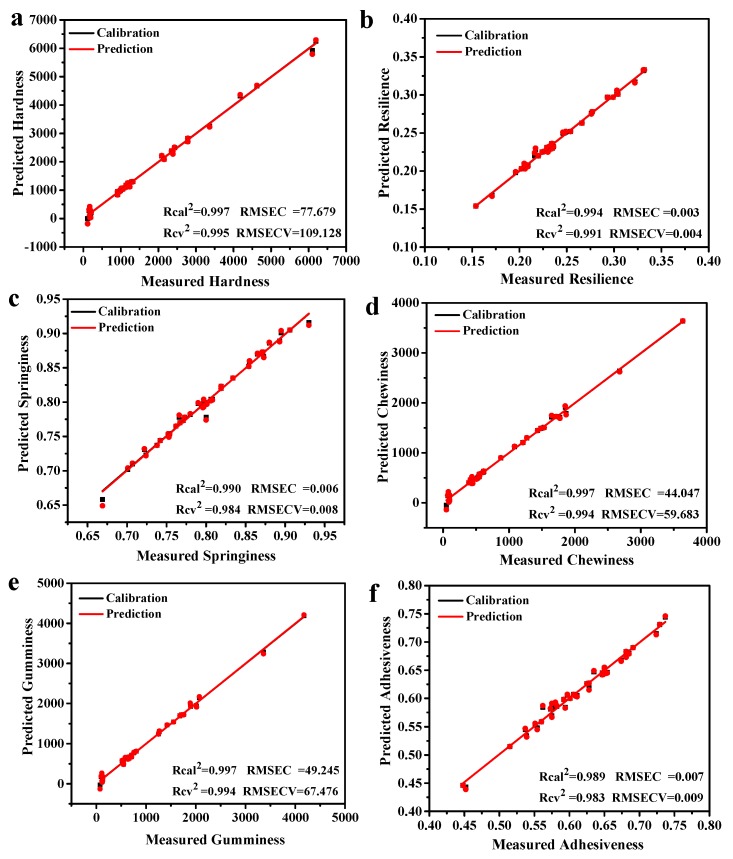
(**a**) Hardness, (**b**) resilience, (**c**) springiness, (**d**) chewiness, (**e**) gumminess, (**f**) adhesiveness in Spanish mackerel samples using LF-NMR data combined with PLS regression.

The PLS regression was then performed to evaluate the correlation between LF-NMR relaxation signal and various texture parameters of mackerel meat using the entire CPMG data. A total of 45 mackerel samples whose texture parameters were known were used to establish the prediction model. As shown in Table 4, the optimal factor number is 7 for the models of hardness, resilience, springiness, chewiness, gumminess and 6 for adhesiveness. The correlation coefficient in calibration (R_cal_^2^) and the correlation coefficient in cross-validation (R_cv_^2^) were more than 0.980, which indicated a good correlation between NMR data and hardness, resilience, springiness, chewiness, gumminess, adhesiveness (Figure 8). The PLS regression was evaluated by a leave-one-out cross-validation. The root-mean-square error of calibration (RMSEC) measures goodness of fit between the testing data and the calibration model, and the root-mean-square error of cross-validation (RMSECV) actually measures performance for unknown cases that are obtained among the calibration cases. The values of RMSECV are less than two-fold RMSEC, and the corresponding residual prediction deviation (RPD) values in the range between 69.282 and 119.389. This indicated that there was good reliability of the models developed for hardness, resilience, springiness, and adhesiveness, and the LF-NMR could be potentially used to nondestructively monitor physical attribute change of samples during boiling, steaming, roasting and frying processes. 

To further evaluate the established PLS regression model for texture parameter prediction, a total of 20 texture-unknown mackerel samples were used to verify accuracy of the PLS models (Table 5). All CPMG relaxation data from the fish samples by LF-NMR were input into the prediction models to estimate the Spanish mackerel texture parameters before and after cooking. As for the verification samples of mackerel meat, the hardness recoveries were in the range of 83.47%–102.91%, and the coefficient of variation (CV) was within 1.27%–4.86%. Recoveries were 100.32%–101.52% for resilience, and CV was 0.23%–1.42%, respectively, whereas recoveries were 99.22%–100.66% for springiness and CV was 0.28%–1.68% by the PLS regression measurements. A relatively larger changing range of recoveries was found for chewiness (76.38%–101.96%) and gumminess (78.34%–102.36%), respectively, with CV of chewiness (1.81%–3.6%) and gumminess (1.67%–3.58%). The control samples before food processing showed lower recoveries for chewiness (76.38%) and gumminess (78.34%). In contrast, the fried fish sample showed a relatively higher recovery (113.57%) for adhesiveness as compared with those (100.18%–100.68%) of control, boiled, steamed and roasted samples. The CV of adhesiveness was in the range of 0.15%–2.17%. Overall, the recoveries for the vast majority of mackerel samples were more than 90% and less than 105%, and the CV was less than 4.86%, suggesting good reliability and accuracy for the PLS regression models.

## 4. Conclusions

In this work, the proton migration of Spanish mackerel before and after boiling, steaming, roasting and frying was characterized and compared by low-field NMR and its relationship to the physical attributes was studied. A significantly reduced proton mobility and declined freedom of the protons were observed, and the LF-NMR and MRI provided a more intuitive approach in monitoring the moisture change during the cooking processes. Different cooking losses and similar WHCs were found for the mackerel sample after the treatment of boiling, steaming, roasting and frying. Cooking resulted in a significant increase of total carbonyl content and TBARS, and a decrease of free thiols and surface hydrophobicity of the fish sample. Significant structural changes were found by the CD and cryo-SEM analysis. The correlation coefficient values of R_cal_^2^ and R_cv_^2^ were more than 0.980, suggesting good correlation between LF-NMR data and hardness, resilience, springiness, chewiness, gumminess, and adhesiveness. Moreover, the PLS regression models exhibited good recoveries and relatively small CV, demonstrating their good reliability and accuracy in predicting texture parameter for mackerel samples. 

## Figures and Tables

**Table 1:** ^1^H low-field nuclear magnetic resonance (LF-NMR) parameters of *T*_2_ relaxation times and peak areas (*A*) of Spanish mackerel before (control) and after boiling, steaming, roasting and frying.

Treatment	*T*_21_ (ms)	*T*_22_ (ms)	*T*_23_ (ms)	*A*_21_(g^−1^)	*A*_22_ (g^−1^)	*A*_23_ (g^−1^)
Control	1.01 ± 0.05^b^	51.04 ± 1.99^a^	429.96 ± 42.12^b^	526.50 ± 102.29^a^	9577.17 ± 682.30^a^	180.82 ± 57.56^d^
Boiling	0.85 ± 0.08^b^	30.21 ± 1.56^b^	533.10 ± 56.36^a^	341.55 ± 15.43^c^	5667.80 ± 281.15^b^	546.17 ± 227.15^c^
Steaming	0.77 ± 0.09^b^	26.60 ± 0.46^c^	275.61 ± 14.37^c^	307.60 ± 15.74^c^	5261.56 ± 198.75^b^	843.55 ± 117.85^b^
Roasting	0.66 ± 0.13^b^	18.01 ± 1.57^d^	228.71 ± 20.67^d^	355.09 ± 42.90^c^	4162.73 ± 891.11^c^	299.38 ± 126.77^d^
Frying	1.44 ± 0.43^a^	17.43 ± 2.50^d^	142.94 ± 16.45^e^	470.28 ± 100.14^b^	1707.53 ± 399.69^d^	1113.33 ± 235.26^a^

Different letters in a column indicate significant differences (*p* < 0.05).

**Table 2:** The content of total carbonyl, free thiols, surface hydrophobicity and thiobarbituric acid reactive substances (TBARS) in Spanish mackerel muscle before (control) and after boiling, steaming, roasting and frying.

Oxidation Indexes	Treatments				
	Control	Boiling	Steaming	Roasting	Frying
Total carbonyl (nmol/mg)	0.11 ± 0.05^b^	0.12 ± 0.01^b^	0.12 ± 0.01^b^	0.17 ± 0.02^b^	0.27 ± 0.08^a^
Free thiols (10^2^ nmol/mg)	4.62 ± 0.42^a^	1.18 ± 0.13^b^	1.02 ± 0.29^b^	0.87 ± 0.01^b^	0.57 ± 0.17^b^
Surface hydrophobicity	54.89 ± 4.86^a^	19.34 ± 0.18^b^	20.67 ± 3.52^b^	15.76 ± 0.03^b^	14.88 ± 0.69^b^
TBARS (mg/kg)	0.17 ± 0.01^c^	0.26 ± 0.00^b^	0.36 ± 0.02^b^	0.64 ± 0.01^a^	0.34 ± 0.06^b^

Different letters in a column indicate significant differences (*p* < 0.05).

**Table 3:** Influences of different cooking methods on texture profile of Spanish mackerel meat.

Processing	Hardness (g)	Resilience	Springiness	Chewiness	Gumminess	Adhesiveness
Control	177.01 ± 11.50^d^	0.23 ± 0.02^b^	0.76 ± 0.03^b^	87.6 ± 9.76^d^	115.78 ± 10.28^d^	0.65 ± 0.02^a^
Boiling	1040.98 ± 120.72^c^	0.24 ± 0.03^b^	0.79 ± 0.03^b^	493.22 ± 84.62^c^	624.36 ± 94.01^c^	0.60 ± 0.03^b^
Steaming	996.51 ± 128.69^c^	0.22 ± 0.01^b^	0.80 ± 0.03^b^	459.91 ± 70.94^c^	576.04 ± 81.92^c^	0.58 ± 0.03^b^
Roasting	2399.01 ± 222.93^b^	0.30 ± 0.04^a^	0.87 ± 0.02^a^	1430.15 ± 235.32^b^	1643.56 ± 263.85^b^	0.68 ± 0.07^a^
Frying	6561.44 ± 623.91^a^	0.25 ± 0.04^b^	0.85 ± 0.05^a^	3255.96 ± 464.38^a^	3826.61 ± 349.78^a^	0.59 ± 0.07^b^

Different letters in a column indicate significant differences (*p* < 0.05).

**Table 4:** Results of partial least squares (PLS) regression models for predicting texture parameters using the entire Carr–Purcell–Meiboom–Gill (CPMG) relaxation data of Spanish mackerel subjected to different cooking methods.

	Calibration			Validation		
	PLS Factors	R_cal_^2^	RMSEC	R_cv_^2^	RMSECV	RPD (%)
Hardness (g)	7	0.997	77.679	0.995	109.128	119.389
Resilience	7	0.994	0.003	0.991	0.004	110.067
Springiness	7	0.990	0.006	0.984	0.008	69.282
Chewiness	7	0.997	44.047	0.994	59.683	97.453
Gumminess	7	0.997	49.245	0.994	67.477	100.154
Adhesiveness	6	0.989	0.007	0.983	0.009	84.817

R_cal_^2^ = Coefficient of determination in calibration; RMSEC = Root mean square error of calibration; R_cv_^2^ = Coefficient of determination in cross validation; RMSECV = Root mean square error of cross validation; RPD = Residual prediction deviation.

**Table 5:** PLS regression model results for predicting texture parameters with the CPMG relaxation data of Spanish mackerel subjected to different cooking methods.

Texture Parameters	Treatment	Predicted Value	Measured Value	Recovery (%)	CV (*n* = 4, %)
Hardness	Control	229.61 ± 7.56	191.84 ± 14.07	83.47 ± 0.04	4.86
	Boiling	1310.50 ± 64.63	1256.00 ± 57.68	95.87 ± 0.02	2.38
	Steaming	1106.50 ± 41.49	1100.75 ± 42.19	99.52 ± 0.03	3.23
	Roasting	2317.50 ± 21.46	2384.75 ± 26.59	102.91 ± 0.01	1.27
	Frying	3838.50 ± 784.29	3761.50 ± 824.16	97.76 ± 0.02	1.62
Resilience	Control	0.23 ± 0.00	0.23 ± 0.00	100.32 ± 0.01	0.89
	Boiling	0.26 ± 0.01	0.26 ± 0.01	100.58 ± 0.00	0.23
	Steaming	0.23 ± 0.00	0.23 ± 0.00	101.52 ± 0.01	0.56
	Roasting	0.31 ± 0.02	0.31 ± 0.02	100.83 ± 0.01	0.76
	Frying	0.22 ± 0.01	0.22 ± 0.01	100.38 ± 0.01	1.42
Springiness	Control	0.76 ± 0.01	0.75 ± 0.01	99.22 ± 0.02	1.68
	Boiling	0.80 ± 0.01	0.81 ± 0.00	100.66 ± 0.01	0.98
	Steaming	0.79 ± 0.01	0.80 ± 0.00	100.51 ± 0.01	1.02
	Roasting	0.89 ± 0.01	0.89 ± 0.01	100.00 ± 0.00	0.28
	Frying	0.86 ± 0.01	0.86 ± 0.02	99.27 ± 0.01	1.18
Chewiness	Control	120.60 ± 3.48	92.08 ± 3.06	76.38 ± 0.03	3.33
	Boiling	629.33 ± 28.67	600.49 ± 27.50	95.46 ± 0.03	3.60
	Steaming	518.68 ± 19.97	528.69 ± 17.46	101.96 ± 0.02	1.81
	Roasting	1285.00 ± 91.08	1304.00 ± 94.53	101.49 ± 0.02	2.14
	Frying	1560.00 ± 310.47	1524.50 ± 319.07	97.59 ± 0.02	1.88
Gumminess	Control	155.59 ± 7.23	121.93 ± 8.26	78.34 ± 0.03	3.58
	Boiling	750.74 ± 44.10	734.73 ± 30.12	97.97 ± 0.03	3.30
	Steaming	643.96 ± 19.80	651.91 ± 9.37	101.29 ± 0.03	2.70
	Roasting	1638.50 ± 93.23	1676.75 ± 88.44	102.36 ± 0.02	1.72
	Frying	1858.75 ± 319.09	1816.25 ± 322.34	97.64 ± 0.02	1.67
Adhesiveness	Control	0.64 ± 0.01	0.64 ± 0.01	100.40 ± 0.01	1.32
	Boiling	0.63 ± 0.01	0.63 ± 0.02	100.44 ± 0.00	0.20
	Steaming	0.59 ± 0.02	0.60 ± 0.02	100.68 ± 0.00	0.15
	Roasting	0.67 ± 0.02	0.68 ± 0.02	100.18 ± 0.01	0.71
	Frying	0.54 ± 0.01	0.62 ± 0.01	113.57 ± 0.02	2.17

CV: coefficient of variation.
